# Can Graphene Pave the Way to Successful Periodontal and Dental Prosthetic Treatments? A Narrative Review

**DOI:** 10.3390/biomedicines11092354

**Published:** 2023-08-23

**Authors:** Alina Mihaela Apostu, Irina-Georgeta Sufaru, Oana Tanculescu, Simona Stoleriu, Adrian Doloca, Alice Arina Ciocan Pendefunda, Sorina Mihaela Solomon

**Affiliations:** 1 Odontology-Periodontology and Fixed Prosthodontics Department, Faculty of Dental Medicine, “Grigore T. Popa” University of Medicine and Pharmacy, 700115 Iasi, Romania; 2 Department of Preventive Medicine and Interdisciplinarity, Faculty of Medicine, “Grigore T. Popa” University of Medicine and Pharmacy, 700115 Iasi, Romania

**Keywords:** antimicrobial properties, dental implants, graphene, graphene derivates, periodontal treatment, prosthodontic treatment

## Abstract

Graphene, as a promising material, holds the potential to significantly enhance the field of dental practices. Incorporating graphene into dental materials imparts enhanced strength and durability, while graphene-based nanocomposites offer the prospect of innovative solutions such as antimicrobial dental implants or scaffolds. Ongoing research into graphene-based dental adhesives and composites also suggests their capacity to improve the quality and reliability of dental restorations. This narrative review aims to provide an up-to-date overview of the application of graphene derivatives in the dental domain, with a particular focus on their application in prosthodontics and periodontics. It is important to acknowledge that further research and development are imperative to fully explore the potential of graphene and ensure its safe use in dental practices.

## 1. Introduction

Dental diseases are a worldwide concern due to their high prevalence and incidence [1]. Moreover, these conditions lead to functional local and loco-regional disorders, affecting the patient’s systemic status and quality of life. Periodontal disease and edentulism occupy an important place within these conditions.

Periodontitis is an infectious-inflammatory disease that, as it progresses, leads to the destruction of the tissues that support the tooth on the dental arch, which may result in tooth loss. In addition, periodontitis has been associated with the negative evolution and aggravation of some systemic conditions and diseases, such as diabetes mellitus [2], cardiovascular diseases [3], rheumatoid arthritis [4,5], chronic kidney disease [6], or inflammatory bowel disease [7].

Edentulism represents a complex, impairing condition with profound implications for performing functions such as mastication, swallowing, phonation, and aesthetics [8]. Edentulism has multiple complications on a local level (e.g., teeth migrations, residual ridge resorption, damage of tooth-supporting tissues, and occlusal problems) [9,10], a locoregional level (temporomandibular dysfunction, masticatory muscle disorder, and craniomandibular disorder) [11,12,13], and a general level (nutritional deficiency, increased risk for certain systemic diseases, mental health impairment, and poor quality of life) [14,15]. Due to its complex and intricate evolution, it requires an integrated treatment plan using biocompatible and biomechanically suited materials to withstand the biological and mechanical conditions of the oral environment.

Thus, establishing effective dental treatment as well as identifying appropriate therapeutic methods is a priority. Dental technology constantly evolves, and new materials have been developed that respond favorably to mechanical stress and oral bacterial conditions. In this regard, nanomaterials have been emphasized as biomaterials with superior physical, chemical, and biological properties [16].

Nanotechnology is a prerogative of the 21st century and impacts the medical field. Any material used for medical purposes, especially those that have long-term contact with the human body, must have specific biological and mechanical properties, such as not interfering with the host environment, not allowing the growth of bacteria on its surface, and not initiating a local inflammatory response.

Graphene fulfills these characteristics, excelling in antibacterial properties and gaining ground in most medical fields, especially dentistry. The antimicrobial effect of graphene-based materials (GBMs) is one of their most exciting properties. This effect is attributed to their physical interaction mechanism with bacteria, which is likely to prevent the development of antimicrobial resistance (AMR) [17,18]. Unlike antibiotics, which interfere with these processes, this mechanism is independent of bacterial activities such as RNA, protein, cell wall synthesis, or DNA replication. Furthermore, graphene materials exhibit antimicrobial activity and do not appear to induce long-term secondary resistance [17]. Equally major concerns exist related to the biocompatibility of graphene, the ability of this material to interact and coexist in a biological environment without causing adverse reactions or harmful effects on the human body [19]. The evaluation of the biocompatibility of graphene is essential in the development of its applications in medicine and biotechnology, such as in the field of medical diagnostics, drug therapy, or implantable medical devices. Studies and research are ongoing to understand in detail the interactions between graphene and the cells of living organisms, as well as to develop techniques to functionalize graphene to improve its biocompatibility [20,21,22].

One question arises: What is graphene? Graphene is a carbon-based material, first theorized by PR Wallace in 1947 and obtained by Andre Geim and Konstantin Novoselov in 2004 [23], for which they were rewarded the Nobel Prize in Physics in 2010 [24]). Since then, tremendous efforts have been made to obtain the material on a large scale, and its commercial production began in 2014. However, one of the limitations of using graphene is the difficulty of processing and agglomeration due to the unique carbon component of pure graphene [25].

Chemical modifications have produced graphene derivatives, including graphene oxide (GO) and reduced graphene oxide (rGO). These derivatives are highly versatile and more applicable [26].

Among the properties that make graphene so special are: stability at extremely small dimensions (one atom thick, meaning 100,000 times thinner than hair), high level of cohesion, hexagonal configuration, and being the most robust material known so far—stronger than steel (200 times), with the same density as diamond (four layers of graphene would support an elephant) [27], elastic and flexible, with high thermal and electrical conductivity, transparent, water-resistant, inert, dense (not even helium passes through it), bacteriostatic, very light (1 m^2^ weighs less than 1 mg), and ecological [28].

Many applications of this material have been implemented in various fields, starting with electronics (ultrafast transistors, flexible displays, or LEDs), energy production and storage (batteries, solar panels), aviation (improving the structure of aircraft wings), telephony, and computers [29].

Medicine is another field that promises to be transformed by graphene. Because this material is thin, flexible, and resistant to the saline solutions that are part of living tissues, graphene is ideal for designing bionic devices. Unlike metal devices, which last only a few years in the human body, graphene can be used for life. Moreover, because graphene is an electrical conductor, it could be used to transmit electrical impulses to neurons, allowing paralyzed people to regain control of their limbs following an accident that resulted in spinal cord damage [30]. The exact mechanism could be used to control artificial limbs by using graphene to transmit electrical signals to motors that set them in motion. Graphene has been investigated for its potential integration into diverse 3D technologies. Three-dimensional printing serves to manufacture intricate and customized structures, including prosthetics, implants, and medical devices, along with functional anatomical models [31,32]. The merger between 3D printing and biotechnology, encompassing methods such as laser-assisted bioprinting, inkjet bioprinting, or microextrusion bioprinting, facilitates the creation of intricate cellular structures, such as synthetic organs or tissues. By integrating graphene into bioprinting inks, the viability and functionality of cells can be significantly enhanced [33]. The technology of graphene-based biosensors, which are both biospecific and nanostructured, enables the precise and sensitive detection of biological molecules. Graphene can be functionalized to selectively interact with a range of molecules, thereby establishing a robust platform for early disease diagnosis and medical monitoring [34]. In nanoparticle therapy, functionalized graphene can serve as a vehicle for the targeted and precise delivery of medications or therapeutic agents to specific locations within the human body. This approach holds the potential to amplify treatment efficacy while minimizing undesirable side effects [35].

**Figure 1:**
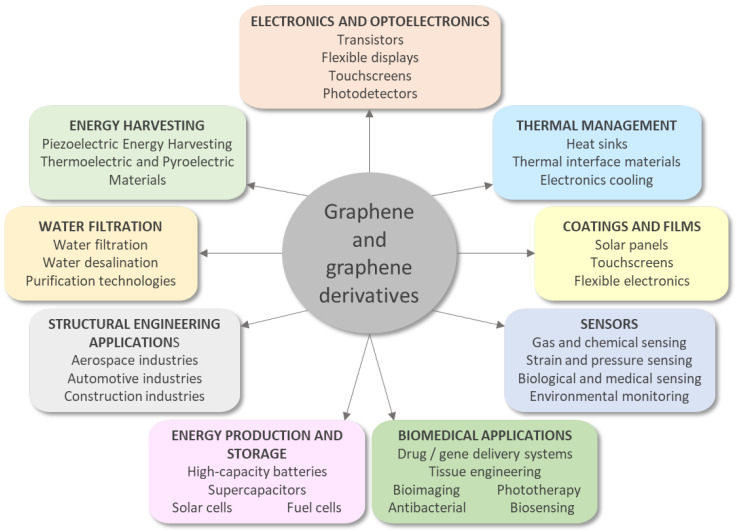
Primary applications of graphene and its derivatives.

The main fields in which graphene and its derivatives are applied are presented in Figure 1.

**Figure 2:**
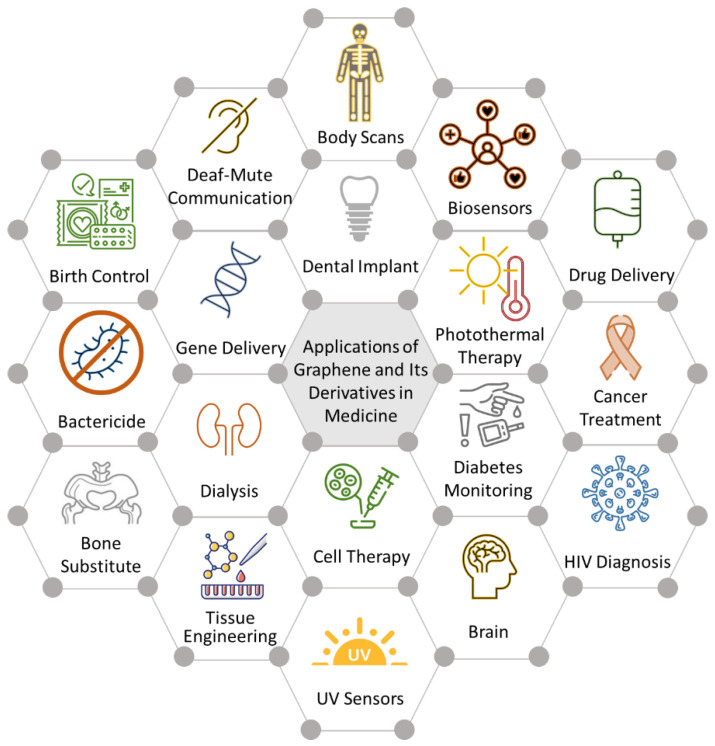
Main applications of graphene and its derivatives in medicine.

Among the medical fields that have explored the potential of graphene and its derivatives is dentistry. Ge et al. and Desante et al. emphasized the existing research void regarding the potential applications of graphene in dentistry, which contrasts with its comprehensive utilization in various other medical domains such as drug delivery, imaging agents, biosensors, and tissue engineering scaffolds [20,36] (Figure 2). However, graphene holds tremendous potential in the dental field. Notably, significant progress has been made in developing biosensors for detecting biomarkers in saliva, including cancer markers, drugs, and bacterial and viral markers. Additionally, graphene has shown promise in drug delivery, teeth whitening, preventing demineralization of enamel and dentin, dental pulp regeneration, treatment of persistent periapical periodontitis [37], and inhibiting fungal growth [28].

Researchers at Princeton University have conducted a noteworthy study where graphene is incorporated onto the tooth surface as a tattoo [38]. As graphene possesses excellent electrical conductivity, it enables the transmission of information about oral fluid microflora using a wireless sensor. This technology can control bacterial plaque and detect diseases early by monitoring changes in the concentration of bacterial metabolism products in respiration and oral fluids [38]. The utilization of graphene-based derivatives has displayed remarkable promise in propelling CAD/CAM technology, particularly in the sphere of high-performance dental restorations [39]. These restorations showcase favorable mechanical attributes and possess the potential for expedited chairside fabrication [40,41].

Our literature research aimed to provide readers with a “broad view” of the potential and applications of graphene-based materials (GBMs) for the treatment of oral conditions, with a specific focus on periodontal disease and edentulism.

## 2. Methodology

A narrative review was conducted aiming to answer the question, “Can graphene and its derivatives contribute to the improvement of periodontal and prosthetic treatment”?

**Figure 3:**
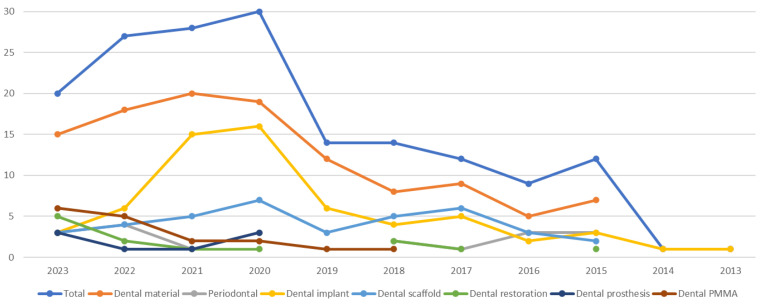
Distribution of selected articles by their publication year (*n* = 168).

**Figure 4:**
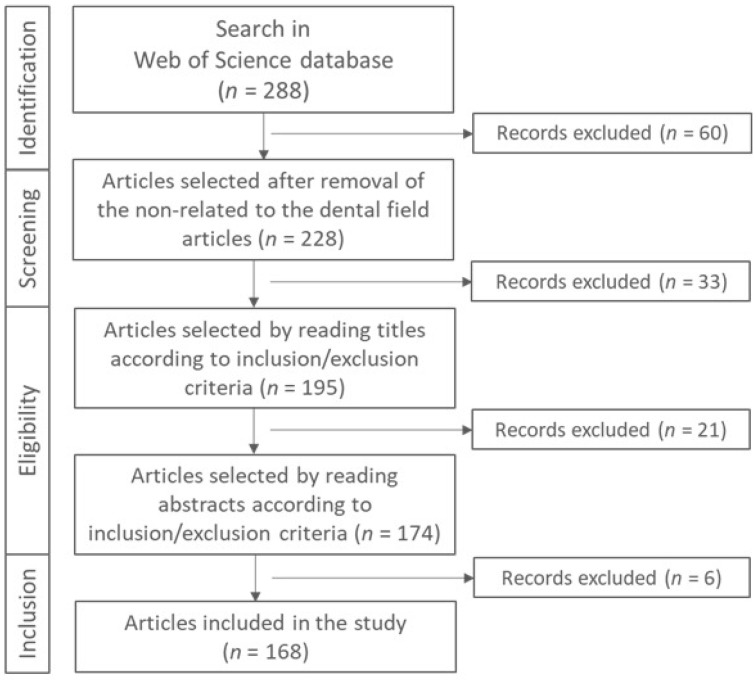
Flow chart of the literature search.

An electronic search was conducted in the Web of Science digital database using MeSH terms and unspecific free-text words with Boolean operators. The final search terms were defined after iterative trial and refinement processes. The strategy employed was as follows: “graphene periodontal” OR “graphene dental prosthesis” OR “graphene dental material” OR “graphene dental implant” OR “graphene dental restoration” OR “graphene dental scaffold” OR “graphene dental PMMA”, within the topic field, without imposing any year restrictions. The most recent search was carried out in July 2023, yielding a total of 288 studies. Review articles, book chapters, editorial materials, and studies unrelated to the medical field were excluded, resulting in 168 studies published in English between 2013 and 2023 (Figure 3). Following an evaluation of titles, abstracts, and full-text articles based on predefined inclusion and exclusion criteria (Table 1), 107 articles were ultimately retained. Figure 4 shows the flow chart of the selection of the studies and the reasons for exclusion. Relevant references from the selected studies were evaluated and incorporated where applicable.

## 3. Graphene Derivatives

**Figure 5:**
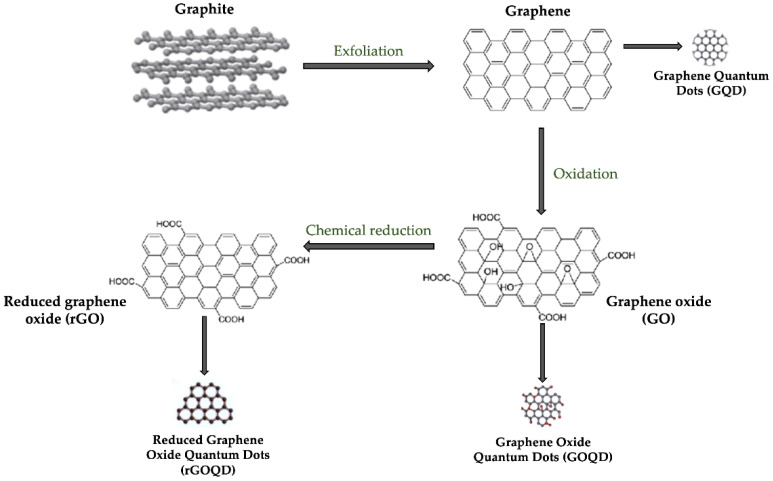
Production of graphene and its derivatives.

Graphene oxide (GO) and reduced graphene oxide (rGO) are the leading derivatives of graphene, as shown in Figure 5. Graphene oxide (GO) was first synthesized by Benjamin Brody in 1859 [42] and can be produced through several available techniques [43,44]; however, the most frequently used method is the Hummers-Offeman method [43,45]. GO has a high specific surface area (890 m^2^/g) [46], and its structure includes oxygen contained in hydroxyl epoxy and carboxyl groups; they contribute to the covalent or non-covalent combination of GO with biomolecules and other nanomaterials [47]. The GO structure increases hydrophilicity and dispersibility in aqueous solutions and other polar solvents [43]. Moreover, GO is biocompatible, can promote cell adhesion and proliferation, and induces directional differentiation of stem cells [48]. GO also proves its antimicrobial properties through various effects, including oxidative stress, cutting edges, and cell capture [45].

Reduced graphene oxide (rGO) is obtained by reducing graphene oxide. This process is ecological, and the resulting product is biocompatible and dispersible [49]. rGO can partially restore the conductivity and absorbance of graphene; it is also more hydrophobic than GO due to the reduction of oxygen-containing groups [45]. rGO is more graphene-like than GO; thus, it exhibits higher photothermal and mechanical properties than GO. These properties enable rGO to be used in anti-cancer photothermal therapy, heat-induced controlled drug release, and antibacterial therapy [50,51].

Graphene quantum dots (GQDs) are produced from graphene oxide. They have strong quantum confinement and photoluminescence properties. These properties make GQDs successful in cell imaging [52].

Nanocomposites that use graphene as a base are created by attaching other active agents to either GO or rGO through covalent or non-covalent bonds [53]. For this purpose, metal ions, oxides, or polymers can bind to graphene, generating products with enhanced properties [45].

Graphene oxide can bond with silver nanoparticles, forming a stable product that favors a more efficient dispersion, which leads to increased antibacterial properties [54]. It has also been shown that the surface cation concentration is higher in this nanocomposite formulation, favoring the effect on the bacterial wall [55].

In addition to metal ions, oxides such as zinc oxide (ZnO), iron oxide (Fe_3_O_4_), or titanium oxide (TiO_2_) have also been used. The addition of ZnO also enhanced the antibacterial effect of GO based on the membrane stress caused by sharp edges on microbial cells [56].

Polymers, such as poly-lysine (PLL) [57], polyvinyl-N-carbazole (PVK) [58], or pluronic [59], have also been added to graphene oxide. These nanocomposites have been shown to exert enhanced antibacterial effects over GO alone [45].

## 4. Antimicrobial Effects

The antimicrobial mechanisms of graphene derivatives are based on both their chemical and mechanical effects [60]. In terms of mechanics, both GO and rGO possess sharp edges that can potentially harm the cell membrane of bacteria [61]. The mechanical factors influencing this effect are the edge density and the contact angle between the sheet and the cell membrane [62]. Research has shown that GO sheets with smaller sizes and smoother edges possess a higher density of edges, leading to a more potent antibacterial effect. This effect has been observed to commence at a contact angle of 37°, with its peak achieved at 90° [62]. Moreover, rGO showed a higher impact in this respect than GO [63].

Another antimicrobial mechanism is based on cellular uptake. Following contact between graphene sheets and bacterial cells, the latter are trapped, isolated from the environment, and without access to nutrients [45]. This effect is enhanced by increasing the lateral dimensions of GO sheets [64]. Thus, a contradiction of elements appears: reduced dimensions are necessary for a more significant cutting effect; in contrast, larger GO sheets can achieve higher cellular uptake.

**Figure 6:**
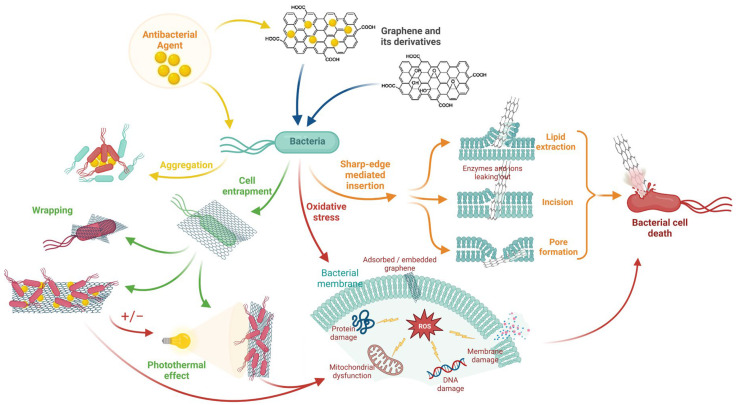
Antibacterial mechanisms of graphene derivatives include cell entrapment, oxidative stress, and sharp-edge-mediated insertion.

Chemically, GO can induce lipid peroxidation in bacteria [65]. This phenomenon is bactericidal because it destroys the cell membrane of microorganisms. Graphene derivatives are believed to produce a high volume of reactive oxygen species (ROS), which can cause oxidative stress in bacteria [66]. The main mechanisms involved in the antibacterial abilities of GO and rGO are presented in Figure 6.

There is a widely held belief that graphene oxide (GO) possesses a greater capacity to generate reactive oxygen species (ROS) and oxidize reduced glutathione than reduced graphene oxide (rGO) [45]. However, it has been observed that both rGO and graphite possess a higher oxidation capacity than isolated GO and graphite oxide [67]. From the existing data, the generated oxidative stress is independent of the size of the GO sheets; however, some reducing agents could diminish this effect [46]. Moreover, the susceptibility of bacterial strains to GO is related to their sensitivity to oxidative stress. It was demonstrated that obligate anaerobes, such as *Porphyromonas gingivalis* or *Fusobacterium nucleatum*, are more susceptible to GO than facultative anaerobes (such as *Streptococcus mutans*) [68].

It is commonly understood that the effects in question are attributable to a combination of mechanical and chemical processes that occur gradually over time. Oxidative stress can facilitate the damage of bacterial membranes by the sharp edges of graphene derivatives through ATP degradation [69].

The antibacterial effect of graphene derivatives on periodontopathogenic bacteria was investigated. Peng et al. compared the antimicrobial activities against *Candida albicans*, *Lactobacillus acidophilus*, *S. mutans*, and *Aggregatibacter actinomycetemcomitans* of rGO and silver (rGp-NS-Ag) composites with those of silver nanoparticles (AgNP) and rGO nanosheets alone. rGp-NS-Ag generated enhanced antimicrobial effects [70]. Treatment of the titanium surface with GO-Ag nanocomposite demonstrated an antibacterial effect on *P. gingivalis* in the percentage of 95.45%, as well as a low rate of bacterial adhesion (4.55%), and showed impacts by data related to microstructures, quantity, cell membrane disruption, bacterial cell apoptosis, and bacterial gene expression [71].

The antibacterial efficacy of GO nanosheets on three distinct bacterial strains, *S. mutans*, *F. nucleatum*, and *P. gingivalis*, was assessed. The approach involved using varying concentrations of nanosheets (20, 40, and 80 μg/mL) to determine their impact on bacterial growth. The results of this study revealed that at a concentration of 40 μg/mL, both *P. gingivalis* and *F. nucleatum* exhibited complete inhibition of growth in the presence of GO nanosheets. However, a reduction of *S. mutans* was observed at a concentration of 80 μg/mL [68]. The mechanism demonstrated by the authors is based on the destruction of the cell wall and membrane by GO, thus leading to plasma leakage.

Graphene-reinforced titanium (Ti-0.125G) was evaluated against the same pathogens (*S. mutans*, *F. nucleatum*, and *P. gingivalis*) [72]. The developed product demonstrated a pronounced inhibitory effect on *P. gingivalis* at 96 h. Moreover, the authors concluded it was broadly effective against multiple pathogens rather than just one strain. It is plausible that the transfer of electrons from bacterial biofilms to the graphene-reinforced titanium element is the underlying mechanism responsible for its anti-bacterial effect. This transfer has been observed to disrupt the bacterial respiratory chain, ultimately reducing microbial viability [72]. Qin et al. proved that GO, followed by brushing, was efficient in biofilm elimination, *P. gingivalis* and *F. nucleatum* included [73].

Wang et al. investigated the antimicrobial properties of graphene-coated Ti-6Al-4V against oral pathogens (*P. gingivalis*, *F. nucleatum*, and *C. albicans*). When coated with graphene, the researchers observed that the Ti-6Al-4V alloy exhibited enhanced resistance against oral pathogens compared to the uncoated Ti-6Al-4V alloy. Moreover, the graphene-coated Ti-6Al-4V alloy generated a higher concentration of ROS in the pathogens tested than the uncoated Ti-6Al-4V alloy [74].

Zinc oxide functionalized graphene oxide polyetheretherketone demonstrated significant antibacterial effects on *P. gingivalis*, *F. nucleatum*, *S. sanguinis*, and *S. mutans*, as well as the prevention of biofilm formation by oxidative stress [75,76]. Research has shown that the implementation of DNA-aptamer-nanographene oxide can also result in the production of reactive oxygen species on *P. gingivalis*, ultimately leading to bactericidal effects [77].

Moreover, Gao et al. demonstrated that graphene oxide coated with mineralized collagen inhibited the bacterial growth of *F. nucleatum* and *P. gingivalis* and disrupted the membrane permeability of free bacteria [78]. Miyaji and colleagues have developed a novel technique for coating with graphene oxide (GO) and cationic surface-active agents that possess antimicrobial properties. Their efforts have resulted in the successful inhibition of oral pathogen growth, which has been observed to endure for up to one week when exposed to water. Furthermore, the antibacterial efficacy of the product can be readily restored through reapplication. Hence, new developments can ease the fight against oral pathogens by means such as a simple mouth rinse with antimicrobial cationic surface-active agents after coating the teeth with GO [79].

Pourhajibagher et al. demonstrated that curcumin-coupled GQDs suppress biofilm formation capacity by 76% for periodontopathogenic bacteria *A. actinomycetemcomitans*, *P. gingivalis*, and *Prevotella intermedia*, as shown by inhibition of biofilm genes (rcpA, fimA, and inpA). The antibacterial mechanism involved is based on the generation of ROS [80]. Trusek and Kijak used bromelain as a releasing enzyme in a mixture with graphene oxide and amoxicillin as a potential drug delivery system for periodontal diseases. The resulting product released drug molecules, inhibiting the growth of bacterial strains sensitive to the antibiotic. The authors also support the advantage of a drug with controlled release, determined by the chosen enzyme concentration [81]. The main studies that focused on the antibacterial effects of graphene derivatives are found in Table 2.

## 5. Implant Surfaces and Osseointegration

Favorable osseointegration for ceramic and titanium implants and their alloys is still a major challenge in implant-prosthetic therapy. To increase the success and survival rate of implants while reducing potential complications following their placement, treatment of bioinert implant surfaces with various materials has been attempted, aiming for enhanced osseointegration through antimicrobial activity and functionalization of the tissue-implant interface. This interface is the site of interaction with the surrounding tissues where all the osseointegration processes occur—inflammatory reactions, cell recruitment, adsorption of proteins, or biofilm formation [95].

Therefore, graphene is utilized in implants due to its exceptional attributes, including high biocompatibility, physical interaction with biomolecules such as proteins, enzymes, or peptides [96], effective stimulation and differentiation of stem cells [97], long-term durability [98], a highly specific surface area that enables subsequent bioactivity [99], improved wear resistance [100], and enhanced toughness [101].

Several techniques, such as chemical vapor deposition [102], plasma treatment [103], electrophoretic deposition method [104], solution spray, dip-coating [20], or wet and dry transfer [105], were used to coat the zirconia and titanium substrates with graphene oxide-based material. GO treatment of inert surfaces improves mechanical properties and promotes cell adhesion and proliferation, which are facilitated by hydrophilic functional groups (such as hydroxyl or carboxyl) [26,106].

### 5.1. Titanium Implants

GO-coated titanium implants stimulated cell proliferation, increased alkaline phosphatase (ALP) activity levels and gene expression levels of osteogenesis-related markers, and promoted BSP, Runx2, and OCN protein expression [107]. Moreover, it was shown that with the increase in thickness of the graphene oxide layer, the ALP-positive areas improved and the mineralization of the extracellular matrix increased [108]. However, the first developed graphene-based coatings did not have a three-dimensional morphology, an essential aspect of the osseointegration process. Thus, the entire group led by Qiu developed the first 3D porous coating based on Go and rGO on pure titanium plates, products that demonstrated a high osteoinduction capacity and biocompatibility [109]. In their observations, Li et al. found that coating titanium with GO resulted in greater new bone mass and fewer gaps between the implants and peri-implant bone tissue [110].

The findings of Cao et al. indicate that the growth of human gingival fibroblasts on TiO_2_ nanotubes resulted in significant improvements in various cellular functions, including proliferation, adhesion, migration, and the expression of genes related to adhesion. These enhancements are critical for achieving successful soft tissue sealing [111].

In their research, Gao and his team employed a sandwich-structured dental implant coating featuring graphene oxide encapsulated within mineralized collagen. This innovative structure demonstrated antibacterial properties and improved adhesion, cytoskeleton organization, and proliferation of human gingival fibroblasts, ultimately leading to superior soft tissue sealing [78].

Enrichment of implant surfaces with GO and bioactive proteins was also attempted. Bone morphogenetic proteins (BMPs) are a class of proteins that can remarkably trigger bone growth. Among them, BMP-2 stands out as a potent factor that facilitates the differentiation of stem cells into bone cells, thereby augmenting the integration of implants by encouraging bone regeneration in the region between the implant and the recipient site [112]. The implant surface was treated with graphene oxide, which was used as a carrier for BMP-2 and substance P [113]. Although no differences in substance P release were observed between the Ti and GO/Ti groups, the release of BMP-2 from Ti/GO was slow for 14 days. In the absence of GO treatment, the release of BMP-2 content occurred within the initial 24 h on the titanium surface [113].

A study conducted by Ren et al. investigated the impact of GO and rGO dexamethasone-loaded titanium foils, specifically DEX-GO-Ti and DEX-rGO-Ti, on the proliferation and osteodifferentiation of rat bone mesenchymal stem cells (rBMSCs). The study results showed that DEX-GO-Ti significantly enhanced cell proliferation. At the same time, rBMSCs cultivated on DEX-GO-Ti demonstrated elevated expression levels of calcium, proteins, and mRNA, which are closely associated with osteogenic differentiation [114].

Titanium implant abutment surfaces treated with minocycline hydrochloride (MH)-loaded graphene oxide films were placed in a canine model of peri-implantitis [115] and compared with Ti, MG/Ti, and GO/Ti surfaces. Analyzing radiographic and micro-CT data, it was found that the Ti and MH/Ti groups displayed a more significant amount of marginal bone loss. GO/Ti group exerted little bone loss, and the bone loss in MH/GO/Ti group was negligible. Moreover, higher concentrations of neutrophils were found in Ti and MH/Ti groups, and almost none of the neutrophils could be observed on GO/Ti and MH/GO/Ti. In the last cases, also lots of osteocytes were found [115].

### 5.2. Zirconia-Based Implants

If the literature abounds in research on improving the surface condition of titanium implants with graphene-based materials, there are fewer studies on the association of these materials with zirconia-based implants [116]. Zirconia ceramics (ZrO_2_) are attractive due to their mechanical, physical, and high chemical and thermal stability, combined with the absence of corrosion or toxicity, leading to decreased peri-implant inflammatory reactions [117] and high aesthetic outcomes. Research has focused on two directions: the addition of graphene-based nanomaterials in the zirconia coating [20,100,118,119] and the uniform incorporation of graphene-based nanomaterials into the zirconia ceramics [120,121,122,123,124,125,126,127,128,129].

Two groups of graphene-based 2D nanomaterial (GBN) fillers for ceramic composites can be distinguished depending on the number of graphene sheets. Graphene nanoplatelets (GNP) have more than ten layers and a thickness lower than 100 nm, while multi-layered graphene (MLG) has fewer than ten layers. The latter are divided into two classes: reduced graphene oxide (rGO) and few-layer graphene (FLG), with two to about five layers [130]. Graphene sheets arranged as coaxial tubes with a nanoscale internal diameter are named carbon nanotubes (CNT). Carbon nanotubes are available in two distinct forms, namely single-wall (SWCNTs) and multiple-wall (MWCNTs) [131]. Both graphene sheets and carbon nanotubes do not disperse well in their pure state, proving excess free surface energy results in instability, generating agglomeration [132] and folding of the layers [133] due to van der Waals forces [134]. Combining zirconia and GBN has been shown to enhance material toughness via various mechanisms, including but not limited to graphene pullout, bridging, crack deflection, and crack branching [130]. The buildup of filler material may lead to the development of stress concentration zones, which can considerably compromise the mechanical strength of the material [131].

As for coating materials for zirconia surfaces, few attempts were made. Kou et al. produced functionalized multi-walled carbon nanotubes (fMWCNTs) that improved Saos-2 cell attachment by increasing the surface roughness of coated zirconia-based ceramic surfaces [118]. Furthermore, both SWCNTs and MWCNTs have proven to have powerful inhibitory effects against a wide range of microorganisms, even after a short exposure time [135,136,137]. The broad-spectrum antibacterial activity of CNTs is explained by their “nano-darts” behavior that pierces bacterial membranes [135], which is dependent not only on CNT composition, geometry, surface modification, and intrinsic properties but also on type and morphology of bacteria, mechanical properties of cell surfaces [135,138], and growth state [139,140].

Li et al. produced a zirconia/graphene nanosheets (ZrO_2_/GNs) composite with a homogeneous distribution of GNs in the ZrO_2_ matrix using the plasma spraying technique [100]. The GN additives enhanced the tribological performance through wear resistance improvement and reduction of friction coefficient.

Desante et al. combined the osteogenic properties of GO with bioinert zirconia implants, employing the dip-coating technique to achieve a thin, homogenous, hydrolytically stable, and mechanically stable GO film on silanized ceramic substrates [20]. The stability of a GO film immobilized in double-distilled water and phosphate-buffered saline for 24 days was tested. Furthermore, the film underwent treatment with a ten-minute sonication in double-distilled water. The cytocompatibility of the GO film was assessed for both mouse fibroblasts and human mesenchymal stem cells. The results demonstrate that the GO film has encouraging characteristics for osteogenic differentiation. The active hydroxyl and carboxyl groups on the GO film are amenable to functionalization by immobilizing biological agents such as growth factors or antibiotics.

As found in Table 3, Morales-Rodriguez et al. produced yttria-stabilized zirconia (YSZ) with a few commercial layers of graphene (FLG) and YSZ with exfoliated graphene nanoplatelets (e-GNP) [130]. One vol% of multi-layered graphene was very effective in reducing hydrothermal degradation. Moreover, the composite incorporating e-GNP, due to its uniform dispersion within the matrix, effectively restricted grain growth and slowed the propagation of the transformation front into the ceramic material. The increase in resistance to hydrothermal aging by reducing the grain size of the zirconia negatively affects the fracture toughness of the Y-TZP ceramics [141]. Yet the incorporation of GBN can improve fracture toughness [126,130].

A homogeneous precursor powder must be obtained to produce zirconia-based graphene-containing composites, which are then compacted and sintered. The processing route of graphene-based ceramic composites is of paramount importance for the mechanical properties of the final material [142]. In 2020, Smirnov et al. described employing a hydrothermal synthesis technique to produce a ZrO_2_/rGO powder [121]. The process involved the hydrolysis of a ZrOCl_2_ solution, leading to the positive charge of zirconia ions. These positively charged ions were attracted to the negatively charged graphene oxide (GO) sheets through electrostatic forces, resulting in the collection of zirconia ions on the surface of the GO sheets. The characterization techniques employed to analyze the structure of the obtained materials confirmed that the synthesis route assured the successful bonding of zirconia nanoparticles to graphene oxide sheets. It allowed the efficient production of uniform zirconia/graphene nanopowders, both economically and practically.

Lorusso et al. conducted a study aimed at assessing the efficacy of biomaterials derived from polymethylmethacrylate (PMMA) in terms of bone integration, with a specific focus on PMMA infused with graphene (GD-PMMA) [143]. In their experiment, researchers inserted 18 PMMA and 18 GD-PMMA implants into the femoral knee joints of male rabbits. The results showed successful integration of all implants with the bone, but notably, GD-PMMA titanium surface implants exhibited superior osseointegration. The authors recommend further animal studies, both in vitro and in vivo, to explore the potential clinical applications of GD-PMMA in dental implant procedures [143,144].

PEEK material has become increasingly prevalent in dentistry due to its diverse applications. These applications encompass oral implant treatments such as oral implants and implant abutments, restorative dentistry procedures such as crowns, fixed and removable dentures, posts and cores, and maxillofacial prosthetics, as well as other oral applications such as orthodontic wires and retainers and scaffolds for cartilage repair [145]. It is feasible to augment its mechanical characteristics by incorporating supplementary materials such as fibers, carbon nanomaterials, and ceramics to optimize the functionality of PEEK for dental applications. Furthermore, fine-tuning processing techniques and parameters can also enhance its appropriateness.

In recent years, advancements have been made in using materials such as carbon nanotubes (CNTs) and graphene to reinforce PEEK composites. The treatment of the surface of the CF/PEEK composite with concentrated sulfuric acid leads to the creation of a three-dimensional porous network. Notably, an increased CF content results in larger pore sizes within this porous layer. Following treatment with a solution of graphene oxide (GO), the sulfonated material exhibits the development of filamentous GO folds on its surface. It has been observed that the contact angles of all samples increase post-sulfonation. However, the GO functional wrinkles cause a significant reduction in the contact angle of the material, leading to an increase in surface hydrophilicity [146].

**Table 3:** Applications of graphene-based materials coated on dental implants.

Material	Effects	Reference
Functionalized multiwalled carbon nanotubes on zirconia	Improved cell attachment	Kou et al., 2013 [118]
rGO/Dex	Osteogenic differentiation	Jung et al. 2015 [147]
GO	Osteogenic differentiation	Zhou et al. 2016 [107]
rGO/Ti	High hydrophilicity; rough surface; biocompatibility; enhanced ALP activity; collagen secretion; osteogenic differentiation	Qiu et al., 2017 [109]
GO/Ti/Dex	Promoted proliferation; accelerated osteogenic differentiation	Ren et al., 2017 [114]
nGO/PEG/PEI/siRNA	Osteogenic differentiation; osteointegration	Zhang et al., 2017 [148]
Single-layer graphene sheets	Osteogenic differentiation	Ming et al., 2018 [149]
GO/aspirin/Ti	Proliferation; osteogenic differentiation	Ren et al., 2018 [150]
GO/HA/chitosan	Promoted apatite formation	Karimi et al., 2019 [151]
Magnesium alloy with graphene nanoparticles	High cytocompatibility and osteogenic properties	Khan et al., 2019 [152]
GO/chitosan/HA	Osteogenic differentiation	Suo et al., 2019 [104]
GO/Ti	Biocompatibility; osteogenic differentiation	Di Carlo et al., 2020 [153]
GO	Re-osteogenesis	Qin et al., 2020 [73]
GO/Ti	Proliferation; adhesion, osteogenic differentiation, and osteointegration	Li and Wang, 2020 [110]
GO/Zirconia	Osteogenic differentiation	Desante et al., 2021 [20]
rGO nanosheets	Osteogenic differentiation	Lu et al., 2021 [154]
Graphene nanoplatelets and yttria-stabilized zirconia	Resistance to aging	Morales-Rodriguez et al., 2022 [130]
Reduced graphene oxide (rGO)-coated sandblasted	Accelerated healing rate with superior osseointegration	Shin et al., 2022 [155]

ALP: alkaline phosphatase; Dex: dexamethasone; GO: graphene oxide; HA: hydroxyapatite; nGO: nanosized graphene oxide; PEG: polyethylene glycol; PEI: polyethylenimine; rGO: reduced graphene oxide; siRNA: small interfering ribonucleic acid; Ti: titanium.

## 6. Periodontal Tissue Regeneration

Graphene oxide is also used to make barrier membranes and scaffolds for periodontal tissue regeneration. During bone regeneration, a barrier membrane is utilized to prevent the infiltration of epithelial cells into the neo-formation site. This promotes optimal healing and ensures the development of new bone tissue. Moreover, such a membrane must possess several properties, which include biocompatibility, site protection and maintenance, cell occlusion, tissue attachment, and clinical sensitivity [156]. In the last decade, methods for barrier membrane treatments with different osteoinductive and antibacterial substances have been proposed to provide supplementary effects from their use.

A titanium membrane was coated with GO in a study conducted by Radunovic. Besides the antibacterial effect exerted on *S. oralis*, *Veilonella parvula*, *F. nucleatum*, and *P. gingivalis*, the GO-Ti membrane did not produce toxic or inflammatory effects. Moreover, the multiplication of human gingival fibroblasts and osteoblastic promotion were observed [157]. De Marco et al. enriched a collagen membrane with GO, and the product obtained was biocompatible. The authors demonstrated that GO did not leak the bulk solution and changed some membrane features, such as stiffness and adhesion between the membrane and the atomic force microscopy tip [158].

Scaffolds for use in periodontal tissue regeneration that have been investigated were made of materials including collagen [159], poly(3-hydroxybutyrate-Co-4-hydroxybutyrate) [160], β-calcium phosphate [161], poly-lactic acid [162], poly-glycolic acid [163], polycaprolactone [164], or chitosan [165]. An ideal scaffold for periodontal tissue engineering must achieve efficient and controlled guidance of stem cell proliferation and differentiation into specific tissue lineages [53].

One of the concerns related to the use of these scaffolds is their mechanical capacity in terms of strength and rigidity [52]. Thus, new materials have been proposed to enrich these scaffolds, including graphene derivatives. GO-reinforced hydroxyapatite (HA) scaffolds were made by spark plasma sintering [166] or sol-gel synthesis and biomimetic treatment [167]. The resulting products have demonstrated resistance and the ability to improve the cell viability of mesenchymal stem cells (MSCs) and induce osteoblastic differentiation. The reinforcement of hydroxyapatite with rGO generated a 203% increase in fracture strength compared to pure HA; moreover, this scaffold stimulated cell proliferation and osteoblastic differentiation [168]. Nie et al. made a three-dimensional porous scaffold from rGO and nanohydroxyapatite; it stimulated cell proliferation, alkaline phosphatase (ALP) activity, and osteogenic gene expression of rat bone MSCs [169].

It was revealed that β-TCP scaffolds, modified with GO, exhibit substantial potential in promoting the growth, activity, and gene expression associated with bone formation in hBMSCs. This is primarily achieved through the activation of the Wingless and Int-1 (Wnt) signaling pathways, which results in significant stimulation of bone growth in laboratory conditions. Furthermore, the use of these scaffolds has demonstrated a noteworthy ability to facilitate bone regeneration in calvarial defects in live subjects [170].

Scaffolds based on chitosan and GO exhibited high water retention capacity, porosity, and hydrophilic nature [171]. Hermenean et al. produced chitosan-GO scaffolds with GO weight in different proportions (0%, 0.5%, and 3%). Scaffolds with three wt% showed highly favorable properties regarding ALP activity in vitro and bone neo-formation in vivo [172].

The association between graphene derivatives and natural polymers, primarily collagen, has also been investigated. Nishida et al. fabricated a GO-coated collagen sponge scaffold and implanted it into the post-extraction alveolus in canine subjects. This scaffold resulted in a fivefold stimulation of bone neoformation compared to the control group [159].

A bilayer composite was synthesized by incorporating silk fibroin into graphene oxide and reduced graphene oxide. This composite has been observed to exhibit beneficial properties in promoting cell proliferation and inducing osteoblastic and cementoblastic cell differentiation in periodontal ligament stem cells [173]. Additionally, it has been shown that implementing this framework resulted in a significant increase in ALP, osterix, and runt-related transcription factor 2 levels, as well as an excess of cementum protein I production [173]. This phenomenon is essential to cell regeneration because most artificial materials require multiple growth factors to promote MSC differentiation. At the same time, this type of scaffold can provide a new stage for cementoblast differentiation without the need for biochemical factors [36,174].

Multi-composite structures have been developed to enhance the mechanical and biological characteristics of scaffolds. Wang et al. synthesized a scaffold of GO and a collagen/nanohydroxyapatite nanocomposite. The high porosity of the obtained product exhibited improved hydrophilic and mechanical properties and outstanding proliferation potential [175].

Scaffolds based on hydroxyapatite, chitosan, and graphene oxide promoted cell adhesion and proliferation and improved osteogenesis in vitro tests [104,176]. The association of chitosan, gelatin, and GO in tissue engineering scaffolds improved protein uptake and differentiation of rat MSCs into osteoblasts [177]. The combination of calcium phosphate cement (CPC) scaffolds with GO-Cu nanocomposites (CPC/GO-Cu) has been observed to enhance the adhesion and osteogenic differentiation of rat bone marrow-derived mesenchymal stem cells (rBMSCs). In addition, this combination has been found to trigger the secretion of vascular endothelial growth factor (VEGF) and BMP-2 proteins. Upon placement in calvarial defects, this scaffold has demonstrated the ability to stimulate both angiogenesis and osteogenesis [161].

A research team led by Zhang has successfully developed a method that utilizes water-soluble graphene oxide-copper (GO-Cu) nanocomposites to coat porous calcium phosphate (CaP) scaffolds. This innovative coating has demonstrated the ability to significantly enhance the adhesion and osteogenic differentiation of rat bone marrow-derived stem cells (BMSCs). Moreover, these scaffolds stimulated angiogenesis and osteogenesis when transplanted into rats with calvarial defects [178]. A GO scaffold was prepared by coating the surface of a 3D collagen sponge scaffold with GO dispersion and implanted into dogs with class II furcation defects. The findings of the study revealed that implementing a GO scaffold yielded a noteworthy upsurge in the generation of periodontal attachment, such as alveolar bone, periodontal ligament-like tissue, and cementum-like tissue, when compared to a scaffold that was left untreated [179].

Graphene-based 3D scaffolds have also been developed and investigated for stem cell growth and differentiation. Crowder et al. employed three-dimensional graphene foams as culture substrates for human mesenchymal stem cells. The cells maintained their viability for one week and strongly expressed the osteogenic markers osteocalcin and osteopontin, indicating their spontaneous osteogenic differentiation without extrinsic growth factors [180]. The study conducted by Park et al. focused on the use of graphene oxide as a coating material to improve the osteogenic differentiation potential of 3D-printed poly(ε-caprolactone) (PCL) scaffolds. Oxygen plasma treatment was utilized to etch the surface of the PCL rapidly and effectively. The proposed scaffolds were observed to enhance cell proliferation and osteogenic differentiation of periodontal ligament stem cells. These findings have potential implications in the realm of tissue engineering and regenerative medicine [181]. Table 4 summarizes the main findings of graphene derivative applications in periodontal tissue regeneration and engineering.

## 7. Restorative Materials

The great potential of graphene derivates has allowed their use in various fields of dentistry. For direct or indirect restorations, restorative dentistry employs composites, adhesives, and cement types with aesthetic properties and high hardness. However, they face limitations due to high polymerization shrinkage and poor antibacterial properties. Graphene nanoplates (GNPs) are utilized as nanofillers in porous and prone-to-dissolution materials such as resins, cement, and adhesives to effectively reinforce commonly used dental composites and exert an anticaries effect.

Adding graphene nanosheets to two different powders of bioactive calcium silicate cement (Biodentine and Endocem Zr) improved bonding time and hardness. However, Endocem Zr experienced significant impairment in bonding properties when adding GNPs. This indicates that while GNPs enhance the physical-mechanical properties of materials, they may not be suitable for all materials in terms of bonding [193]. Incorporating graphene and GO into bioactive materials has improved the differentiation and proliferation of human dental pulp stem cells and periodontal ligament stem cells. This, in turn, can potentially facilitate the regeneration of the dental pulp and periodontal ligament [194].

A graphene variant, Fluorinated Graphene (FG), has been developed and incorporated into glass ionomer cement. In dentistry, FG has emerged as a more desirable option than conventional gray GNPs owing to its visually appealing bright white color, which renders it an excellent choice for aesthetic applications. FG has been used to modify poly(acrylic acid)-based glass ionomer types of cement (GICs), offering advantages in mechanical, tribological, and antibacterial properties. Therefore, the GIC/FG composites exhibit increased Vickers microhardness and compression, flexural strength, and a decreased friction coefficient. This widens the application of glass ionomer cement in restorative dentistry for various procedures, such as restoration of non-carious and carious lesions, class III and class V restorations, and crown cementation. Furthermore, these compounds demonstrate potent antibacterial activity against *Staphylococcus aureus* and *S. mutans* while exhibiting a favorable release rate of fluoride ions [195].

The advantages of GBMs have led to their application in enhancing the properties of adhesive materials [196]. Dental composites replace infected dental tissues and prevent the progression of decay caused by existing bacteria in the region. Adhesive materials promote bonding between the dental composite material and dental hard tissues, sealing the tooth restoration interface for bacteria access. Adhesion to dentine is challenging compared to enamel due to its higher water content and reduced mineralization. The resin-dentin debonding is primarily attributed to hybrid layers that degrade dentin collagen fibrils by activating host-derived matrix metalloproteinases (MMPs). Researchers have developed graphene quantum dots with 1-ethyl-3-(3-dimethyl aminopropyl) carbodiimide (EDC) to inhibit the degradation of collagen fibrils. These dots effectively inhibit collagenase activity and MMPs by covalently linking collagen fibers, thereby reducing the enzymatic hydrolysis of collagen fibers and improving the durability of dental bonding material [197].

GNPs are frequently utilized as fillers in polymer-based dental adhesives owing to their potent antimicrobial and antibiofilm characteristics. These nanocomposites, filled with GNPs, exhibit efficacy in suppressing the activity of *S. mutans* cells while simultaneously preserving their bonding properties [198]. Therefore, GNPs serve as ideal fillers for dental adhesives, maintaining both antibiofilm activity and mechanical performance. The utilization of silver nanoparticles to dope reduced nanographene oxide and graphene nanoplates was investigated. The results indicated that these materials display favorable adhesive properties, which enhance the bonding between resin and dentin. Additionally, the cell viability of the adhesive was determined to be more than 85% [199]. According to alternative research, incorporating graphene oxide and hydroxyapatite in resin-dentin bonds can improve their durability, adhesive properties, and remineralization capabilities [200].

A research team led by Nizami has successfully developed a composite material known as nHAP/MWCNT-GO that exhibits exceptional properties as a shield for dentin. The composite comprises nanohydroxyapatite, multi-walled carbon nanotubes, and graphene oxide. This material forms a surface film that effectively resists acid and minimizes dentin erosion [201].

In addition, graphene oxide (GO) has been modified by incorporating different nanoparticles, such as calcium fluoride, silver, and tricalcium phosphate, to prevent dentin decalcification. GO combined with silver and silver-calcium fluoride exhibited inhibition of *S. mutans*. Furthermore, the composite demonstrated low cytotoxicity, except at higher concentrations of approximately 0.1 w/v% [202].

The application of silane primer significantly influences the bonding of zirconia, with the adhesive layer proving to have low mechanical properties [203]. Incorporating GO sheets into silane primers is a viable option to improve the mechanical properties of the adhesive layer in resin composites bonded to ZrO_2_ [204]. Adding GO sheets significantly enhances the shear bond strength between resin composite and ZrO_2_, improves surface roughness, and slightly increases the water contact angle [152].

## 8. Prosthodontic Restorations

Dentistry currently utilizes various types of medical materials, each with its own advantages and disadvantages. Regarding prosthodontics, there is a great diversity of materials for indirect restorations, especially those used in CAD/CAM systems [205]. Due to graphene’s improved mechanical properties, ease of processing and functionalization, and potential use for dental and biomedical applications, it is of great interest to create new enhanced restorative materials with distinct compositions and microstructures, to understand their physical and mechanical performances, and to anticipate their clinical performance and risks of failure [206].

For the past 80 years, polymethylmethacrylate (PMMA) resin has remained a highly favored material within the realm of prosthetic dentistry. This is particularly true for the manufacturing of complete and removable partial dentures. One of the reasons for this popularity is the material’s ease of manufacture, affordable cost, pleasing aesthetic properties, and low modulus of elasticity. Moreover, PMMA resin also boasts easy repair capabilities, making it a versatile choice for dental professionals. Polymethyl methacrylate (PMMA)-based resins find extensive application in provisional restorations due to their low mechanical properties that prevent their use for permanent restorations, considerable polymerization shrinkage, and poor inhibition of biofilms that restrict their use in permanent restorations [207]. Efforts to enhance the mechanical properties of PMMA resins have yielded successful outcomes through the incorporation of reinforcing phases, including glass and polyethylene fibers [208,209,210]. GFMs have had a spectacular evolution, sustained by recent developments in nanotechnology, with graphene nanofibers and nanosheets being incorporated as a reinforcement phase in several polymers [211,212,213,214], including polymethyl methacrylate (PMMA) based resins [215,216]. Even at low concentrations, GO and rGO solutions appear dark, and pristine graphene absorbs a substantial portion of the white light [217]. This can present challenges when using these materials in restorative and prosthetic applications that rely on superior optical properties.

Azevedo et al. have achieved a remarkable result in the realm of maxillary full arch rehabilitation by introducing graphene oxide into PMMA resin, resulting in complete restoration [218]. Recent research has suggested that the incorporation of GO into PMMA resin may provide advantageous results in prosthetic rehabilitation. No mechanical, aesthetic, or other complications were observed in a study spanning eight months.

A study was conducted by Abad-Coronel et al. to compare the fracture resistance of various materials used in temporary restorations. The materials included PMMA, graphene-modified PMMA (GRA), acetal resin (AR), and polysulfone (PS). The purpose of the study was to evaluate the effectiveness of different materials for temporary restorations and their ability to resist fractures. The materials were employed in the fabrication of a three-unit fixed dental prosthesis (FDP) using a milling process facilitated by a CAD/CAM system. During compression testing, it was observed that PMMA displayed substantially lower values in contrast to other materials, whereas PS exhibited the highest values. Moreover, GRA and AR showed similar values, which were still greater than those of PMMA. These findings suggest that GRA, AR, and PS are feasible options for interim milled restorative materials and can potentially serve as substitutes for PMMA. This study highlights the comparative fracture resistance of various materials utilized in temporary restorations and emphasizes the potential benefits of GRA, AR, and PS in this domain [41].

A study was conducted by Bacali et al. that focused on the incorporation of graphene-silver nanoparticles (Gr-Ag) in PMMA. The study aimed to assess the material’s mechanical properties, hydrophilic abilities, and morphology [219]. According to the findings, it has been discovered that the use of Gr-Ag fillers has significantly influenced the compression parameters, bending strength, and tensile strength of the material. This has resulted in an overall improvement in the mechanical properties of the material when compared to pure PMMA. Another study conducted by Bacali assessed the efficacy of Gr-Ag-modified PMMA in combating bacterial infections as well as its potential toxicity, monomer release, and mechanical properties. The findings indicated that this material demonstrated robust antibacterial properties against several strains of bacteria, including Gram-negative bacteria, *S. aureus*, *Escherichia coli*, and *S. mutans* [94].

The utilization of graphene oxide nanosheets (nGO) by Lee and colleagues has been found to effectively enhance the antimicrobial and adhesive properties of PMMA resin [216]. The findings from the antimicrobial-adhesive test indicate that the groups treated with nGO exhibited superior antimicrobial properties against various microorganisms in comparison to PMMA alone. Furthermore, PMMA with a 2% nGO concentration demonstrated enhanced antiadhesion effects against *Candida albicans* after 28 days of culturing, indicating a positive impact on hydrophilicity. These results suggest that incorporating nGO into PMMA may have potential applications in improving antimicrobial and antiadhesive properties for medical devices and implants.

Adding less than 1% graphene nanoparticles to the component of PMMA improves the physical characteristics and confers antimicrobial activity [220]. The considered mechanism is reducing the amount of residual monomer [220,221] and toxicity and improving the material surface quality [222,223] so that the improved chemical composition of a polymeric surface significantly impacts biofilm formation. Moreover, microbial growth seems to be promoted by residual leakage of unpolymerized monomers [224], and improvement of the polymerization process through incorporating graphene results in a significant anti-adherent effect of graphene nanostructures [39,216]. Although the presumed mechanism is not yet fully known, the hypothesis is supported by Surnova et al., who showed that graphene acts as a catalyst, improving the curing process of an epoxy resin [225]. Table 5 comprises the main studies on graphene derivatives’ applications in direct and indirect dental restorations.

Numerous studies have investigated the intrinsic antimicrobial properties of graphene [239]. These properties are attributed to the physical disruption of microbial cell membranes or walls and the induction of oxidative stress, leading to the inhibition of microbial growth [67,240]. Additionally, graphene films used as coatings can contribute to increased hydrophobicity of surfaces, which is associated with reduced microbial growth [40]. Another mechanism that enhances the antimicrobial effect involves depriving the bacteria of nutrients by cutting off their supply [239]. However, further research is necessary to determine whether the dispersion of graphene can enhance the hydrophobicity of PMMA and consequently affect biofilm formation.

According to previous research, the incorporation of graphene within PMMA did not yield a noteworthy effect on the adhesion of microorganisms. This is attributed to the intricate mechanics of microbial adhesion, which are subject to the topographical features and roughness of the material surface and the very low concentration of graphene present in the material [241].

Research has shown that the incorporation of graphene nanofibers into PMMA resin can significantly improve its mechanical and antimicrobial properties, making it a promising material for CAD/CAM applications. While further research is necessary to fully assess its potential, the findings from previous studies are highly encouraging. The use of graphene nano-reinforced biopolymer in G-CAM discs, designed explicitly for permanent dental structures, offers various benefits. These discs are available in different chromatic crowns, providing a natural aesthetic appearance. Moreover, they address the mechanical, physicochemical, and biological shortcomings associated with other materials currently used in the dental industry. The biopolymer discs nanoreinforced with G-CAM graphene (G-CAM, Graphenano Dental, Valencia, Spain) offer numerous properties for dental structures and meet the requirements to be considered a good material for prosthetic works utilizing CAD/CAM technology [234]. Aati et al. conducted a progressive loading of GNPs into a 3D-printed dental resin, aiming to increase resistance to crack propagation, improve mechanical properties, and induce drug-free antimicrobial efficacy against Candida albicans biofilm. The various properties investigated exhibited noteworthy variations and dependencies based on the GNPs content. Notably, material strength experienced a significant enhancement at lower GNP concentrations (≤0.05 wt%). Concurrently, the introduction of GNPs led to progressive improvements in hardness, elasticity, degree of conversion, and surface roughness, culminating in a GNP content of 0.25 wt%. Furthermore, the self-induced inhibition of *C. albicans* growth displayed a proportional relationship with the GNPs content. The alteration did not trigger a toxic reaction, as its biocompatibility remained within the prescribed range for biomedical devices [236].

## 9. Concerns about the Use of Graphene and Its Derivatives

Despite the increasing research on the antimicrobial properties of graphene-based materials (GBMs), there remain uncertainties regarding the mechanisms underlying their behavior and short-term and long-term effects. GBMs exhibit antibacterial activity independent of antimicrobial resistance (AMR) and do not appear to induce long-term secondary resistance. This unique characteristic makes GBMs suitable for various antimicrobial applications; however, their potential toxicity to the environment and human health needs to be thoroughly understood [242]. Considering the widespread use of graphene in medicine and dentistry, concerns arise regarding its toxicity, mainly due to its nanoscale size, which enables penetration of physiological barriers and accumulation in different body regions. Moreover, there is a lack of understanding regarding the ease of graphene excretion from the body [243]. There is also a need for further knowledge regarding the long-term stability of graphene-based materials. Materials and coatings based on graphene oxide (GO) raise specific concerns, particularly in humid and corrosive microenvironments. The hydrophilic nature of GO can lead to detachment from substrates or leaching from materials. This becomes problematic as these GO-based particles are likely to enter the bloodstream, potentially causing harm to tissues and organs. Therefore, it is crucial to better understand the behavior and long-term stability of biomaterials containing graphene and its derivatives to mitigate these risks [244,245].

As a catalyst in oxidative environments, graphene can penetrate cell membranes through its jagged edges and disrupt normal cellular functions [45,56,61,62,69]. Although graphene is currently employed in drug delivery applications, directly introducing it into the human body, its safety profile and toxicity mechanisms have yet to be fully clarified. Current studies indicate that graphene can induce an inflammatory response, leading to local necrosis, DNA damage, and activation of cellular apoptosis or autophagy [246]. While dentistry does not involve the direct insertion of isolated graphene into the body, weakly bound graphene may unintentionally be released from implant surfaces [247], and disconnected graphene from the surfaces of dental materials can be inadvertently swallowed or inhaled [248]. However, the described injuries are primarily associated with cumulative exposure to graphene concentration, dimensions, surface structure (including sharp edges), and functionalization state [249].

Dziewięcka et al. investigated the cytotoxicity of various graphene oxide nanoparticles based on their structure. They systematically produced and analyzed the particles regarding cell viability, oxidative stress, apoptosis stages, and DNA damage. Biomarkers were correlated with various physicochemical parameters of graphene oxide [250]. The authors concluded that even slight changes in chemical composition or morphology could lead to significant differences in cytotoxicity. A similar conclusion was drawn by Neuss et al. when exploring the impact of different polymers on cytotoxicity [251]. Hence, the cytocompatibility of a specifically designed substrate, such as GO nanoparticles, cannot be predicted, necessitating iterative experiments. The lack of consensus is also evident in in vivo studies, where variations in tested materials and concentrations can profoundly affect the observed outcomes [20].

## 10. Discussion

The stomatognathic system is a highly intricate and sophisticated physiological mechanism that remains in a perpetual state of flux due to countless environmental, supra-, and sub-systemic factors. Its primary function is to maintain a delicate equilibrium between a host of risk and protective factors. Therefore, it is imperative to precisely identify and quantify any factor that disrupts this equilibrium to determine the most efficacious treatment approach. However, the oral cavity is replete with numerous variables that pose a significant challenge to accurate measurement and prediction [13].

The efficacy of dental treatments is contingent upon the biological and biomechanical responses of restorations, which are influenced by a plethora of factors. Within the dental industry, there exists a persistent demand to extend the longevity of dental restorations, driven by patients, public practitioners, and administrative bodies in the public health sector. Despite significant advancements in biomaterials and restorative techniques, absolute therapeutic success cannot be guaranteed. Nevertheless, there are ongoing initiatives aimed at enhancing existing biomaterials and developing novel ones that surpass current standards [210].

Contemporary dental materials have undergone extensive development through advanced research conducted in diverse fields such as materials science, chemistry, physics, and engineering. For example, dental composites, which are used for aesthetically pleasing fillings and restorations, were developed based on composite materials that were initially employed in the aerospace and automotive industries. Furthermore, titanium, which is widely acclaimed for its exceptional properties such as its resistance to corrosion and biocompatibility, has found extensive use in dentistry thanks to research conducted in the aerospace industry. Moreover, restorative materials used in prosthodontics and CAD/CAM technology are the outcome of research conducted in the fields of engineering and IT. Additionally, groundbreaking research in biomaterial science has led to the development of bioactive materials that can facilitate dentin remineralization. The integration of technologies and materials from other fields has enabled the field of dentistry to make significant progress and offer more efficient, durable, and aesthetically pleasing dental treatments.

**Figure 7:**
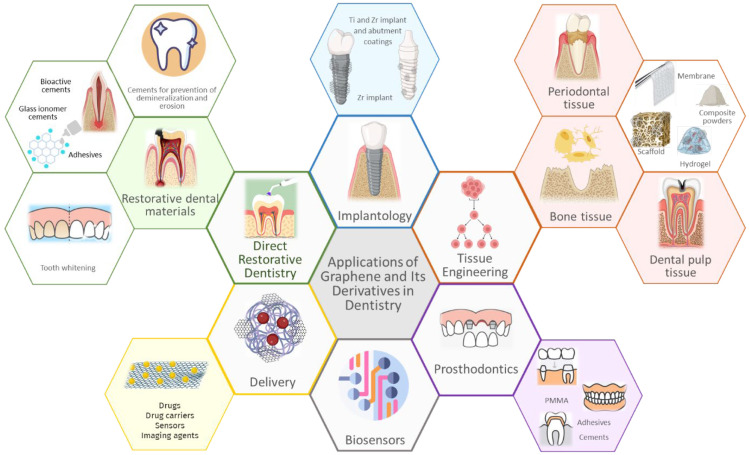
Applications of graphene and its derivatives in dentistry.

The remarkable antimicrobial, chemical, physical, and mechanical attributes of GBMs justify the large interest in their application in the biomedical and dental fields (Figure 7), an interest further supported by the possibility of their cost-effective and reliable production [121].

Numerous factors, namely concentration, size, shape, and surface chemistry, exert a significant influence on the properties of graphene derivatives. It is noteworthy that functionalized graphene oxide and reduced graphene oxide exhibit the most robust antibacterial properties among graphene derivatives [252]. Furthermore, both 2D and 3D graphene derivatives demonstrate the ability to facilitate cell growth and differentiation in the presence of specific chemical reactions with biomolecules.

Titanium- and zirconia-based implants are widely regarded as the optimal choice for dental root replacement due to their exceptional biocompatibility, impressive corrosion resistance, and exceptional long-term performance. However, it is worth noting that titanium does have a relatively low shear strength [253], and zirconia is characterized as bioinert. The mechanical properties of both implant types can be enhanced through surface treatment with graphene derivatives. These nanostructured biological coatings present a multitude of advantages that render them highly attractive for diverse biomedical applications, with the potential to evolve into a pivotal asset in addressing various diseases.

It has been found that graphene and its derivatives positively impact the biocompatibility of materials and promote cell adhesion to the substrate. In comparison to the Gelatin-alginate scaffold, the Gelatin-alginate/Graphene oxide scaffold has exhibited superior compressive strength, enhanced cell attachment and proliferation, amplified expression of osteoblast transcription factors, and elevated activity of alkaline phosphatase [254]. The incorporation of graphene oxide (GO) into films comprising chitosan, polyvinyl alcohol, hydroxyapatite, and gold has been found to result in a significant increase in both porosity and tensile strength. Furthermore, these films exhibit improved antibacterial properties, hemocompatibility, alkaline phosphatase activity, and osteoblast differentiation [192].

Research has shown that a scaffold made from a combination of 3D-printed poly(ε-caprolactone) (PCL) and graphene exhibits improved protein adsorption, cell attachment, and dispersion, as well as increased cell viability and connective and mineralized tissue formation in both laboratory and animal testing. Furthermore, the levels of proinflammatory molecules TNFα- and IL-1β were observed to decrease [255]. In comparison to nanohydroxyapatite on its own, the combination of nanohydroxyapatite and graphene nanoribbons demonstrated a notable augmentation in both alkaline phosphatase and bone neoformation. Furthermore, the suggested product did not result in cellular death even at elevated concentrations [256]. It is noteworthy to highlight that the inclusion of GO in a material has the potential to augment the favorable interactions observed between the cellular component and the material’s surface.

Another study discovered that the inclusion of GO within collagen 3D sponges resulted in a noticeable enhancement of osteoblastic differentiation in vitro and an accelerated rate of new bone formation in vivo [159]. Therefore, graphene and its derivatives have the potential to enhance cytoskeleton development and promote cell adhesion within scaffolds. This information suggests that incorporating graphene-based materials into scaffold design may offer benefits in promoting optimal cell growth and tissue regeneration.

Literature data have demonstrated that GO can have a low cytotoxic effect, resulting in reduced cell viability [257]. It is notable, however, that the response of cells to GO is dependent on the specific type of graphene derivative and the concentration used in the material. Studies have shown that silanized GO [258], starch-(functionalized) reduced graphene oxide nanosheets [259], or hydroxyapatite-zinc-rGO exhibited higher cell viability than GO or rGO alone [260].

In prosthodontics, the conventional technology used for manufacturing resin-based restorations negatively affects the materials’ sustainability and their physical, chemical, and mechanical properties. This is primarily due to factors such as resin shrinkage, residual monomer, and porosity. To address and mitigate these shortcomings, 3D technologies have been adopted in conjunction with the incorporation of graphene additives.

Nevertheless, the utilization of graphene derivatives poses certain challenges, including toxicity and a paucity of comprehensive knowledge concerning their biological characteristics and pathways. These challenges demand further in-depth examination.

Currently, there is a limited amount of research available on the long-term effects of graphene derivatives, which has made their potential toxicity a primary concern. This lack of data has had a significant impact on the widespread use of these biomaterials in clinical settings, and as such, it is crucial to prioritize human safety when considering the use of these materials. To achieve the best possible clinical outcomes, it is necessary to develop solutions to the potential challenges posed by graphene derivatives, including their toxicity and biodegradability. This can be accomplished by developing standardized parameters for their use, as the toxicity and biological features of these materials are inherently linked to their physicochemical properties. Additionally, it is important to note that the effects of graphene derivatives are highly dependent on the dosage and time of exposure, which can significantly impact the potential for detrimental impact on cells through ROS scavenging or oxidative stress.

Our research employed a narrative review approach, a method that, besides its scientific value, is also acknowledged for its inherent limitations within the scientific community. The selection criteria for our study have been thoughtfully established by our team, drawing upon our collective experience. Nevertheless, we acknowledge that this method may be prone to subjectivity, and therefore, we have implemented measures to ensure that only the most reliable sources are utilized. Specifically, we have meticulously curated articles from the Web of Science database. By doing so, we have taken the necessary steps to guarantee the quality of the sources used in our study.

## 11. Conclusions

A comprehensive understanding of the potential use of graphene derivatives within the fields of periodontology and dental prosthodontics was provided. Graphene derivatives have been found to exhibit antimicrobial properties through a combination of mechanical and chemical mechanisms, including cellular uptake, the generation of reactive oxygen species, and sharp-edge-mediated actions. Incorporating derivatives of graphene into coatings for titanium and zirconia-based implants, as well as scaffolds for tissue engineering, has shown promising results in terms of biocompatibility and the formation of new tissue. The physical-mechanical properties of direct and indirect restorations were notably enhanced through the incorporation of various graphene types and concentrations. It has been observed that GO may potentially have a cytotoxic effect that could lead to reduced cell viability. It is important to note, however, that the response of cells to GO is influenced by the specific type of graphene derivative and the concentration utilized in the material.

While we cannot definitively confirm whether graphene is currently viable for successful periodontal and dental prosthetic treatments due to the limitations of the investigated materials, our research highlights potential areas for further exploration. Extensive and rigorous research endeavors are imperative in the domains of implantology and tissue engineering to determine the safety and durability of employing graphene derivatives as a viable course of treatment. We maintain the conviction that the unwavering dedication and persistent efforts of researchers hold the potential to pave the way for the development of reliable, efficacious, and tailored therapeutic interventions, ultimately leading to optimal therapeutic outcomes.

## Figures and Tables

**Table 1:** Inclusion and exclusion criteria of the literature research.

Inclusion Criteria	Exclusion Criteria
English language In vitro studies In vivo studies Research focused on dental biomaterials used for direct and indirect dental restorations, as well as periodontal treatment	Full text is not available Review articles Opinion articles Nonprimary research Studies on graphene materials that are not for oral or dental applications Studies on biomaterials used in dental fields other than restorative and periodontal dentistry

**Table 2:** Antimicrobial activity of graphene-based materials.

Material	Pathogen	Antimicrobial Outcomes	References
GO	*E. coli*	Antimicrobial activity by insertion, edge-cutting, and lipid extraction	Tu et al., 2013 [82]
Ag/GNP	*E. coli*	Antimicrobial activity by cell entrapment	Vi et al., 2018 [83]
Ag-rGO	*E. coli*	Antimicrobial activity by cell entrapment, cell membrane damage, oxidative stress, and the bactericidal action of Ag^+^	Moghayedi et al., 2017 [84]
GMgO-Ag	*E. coli*	Antimicrobial activity caused by membrane damage	Zhang et al., 2016 [85]
GQD	*E. coli*	Antimicrobial activity by ROS generation, photoexcited killing, and cell membrane damage	Ristic et al., 2014 [86]
GO	*E. coli*	Antimicrobial activity	Aunkor et al., 2020 [87]
GO and rGO-poly(dopamine)	*S. aureus*	Antimicrobial activity by cell membrane damage, ROS generation, and electron transfer	Jia et al., 2016 [88]
GO-AgNPs	*S. aureus*	Antimicrobial activity by cell membrane damage and ROS generation	Jaworski et al., 2018 [89]
PLGA/chitosan/GO/AgNPs	*S. aureus*	Antimicrobial activity by catalytic oxidation by silver, cell membrane damage, and ROS generation	De Faria et al., 2015 [90]
rGO/Ag	*S. mutans*	Antimicrobial activity by cell entrapment and the bactericidal action of Ag^+^	Wu et al., 2019 [91]
GO	*S. mutans*	Up to 80% antimicrobial activity	Yu et al., 2020 [92]
Nano-graphene oxide with antisense vicR RNA plasmid	*S. mutans*	Reduced virulent-associated gene expressions, suppressed biofilm aggregation, and inhibited EPS accumulation	Wu et al., 2020 [93]
GO/AgNPs	*C. albicans*	Antimicrobial activity by cell membrane damage and oxidative stress	Jaworski et al., 2018 [89]
GO	*E. coli* *S. aureus*	Antimicrobial activity by disruption of bacterial cellular membranes	Farid et al., 2018 [69]
Nanographene oxide	*P. gingivalis*	Biofilm and bacterial metabolism reduction	Pourhajibagher et al., 2022 [77]
GO	*S. mutans* *F. nucleatum* *P.gingivalis*	Antimicrobial activity by cell membrane damage	He et al., 2015 [68]
rGNs/Ag	*C. albicans* *L. acidophilus* *S. mutans* *A. actinomycetemcomitans*	Higher antimicrobial properties than R-GN and AgNPs alone	Peng et al., 2017 [70]
Ti/GO/Ag	*S. aureus* *S. mutans* *P. gingivalis*	Antimicrobial activity by bacterial cell shrinking, perforation, breaking, and bursting	Jin et al., 2017 [71]
GO	*S. mutans*; *P. gingivalis*; *F. nucleatum*	Elimination of residual bacteria and inhibition of biofilm reformation	Qin et al., 2020 [73]
G/AgNp	*S. aureus* *S. mutans* *E. coli*	Antibacterial activity	Bacali et al., 2020 [94]
Ti/0.125G	*S. mutans* *F. nucleatum* *P. gingivalis*	Suppressed bacterial growth	Wei et al., 2021 [72]
PEEK/GO	*S. mutans* *F. nucleatum* *P. gingivalis*	Bacterial inhibition	Guo et al., 2021 [75]
Ti/6Al/4V	*C. albicans* *P. gingivalis* *F. nucleatum*	Bacterial inhibition by ROS generation	Wang et al., 2022 [74]
GQD	*A. actinomycetemcomitans* *P. gingivalis* *P. intermedia*	Bacterial inhibition by ROS generation	Pourhajibagher et al., 2019 [80]

Ag: silver; G: graphene; G–AgNp: graphene silver nanoparticles; GQD: graphene quantum dots; GO: graphene oxide; MgO: magnesium oxide; Np: nanoparticles; PDA: poly-dopamine; PEEK: Poly-ether-ether-ketone; PLGA: poly(lactic-co-glycolic acid); rGNs: reduced graphene nanosheets; rGO: reduced graphene oxide; RNA: ribonucleic acid; ROS: reactive oxygen species; Ti: titanium.

**Table 4:** The potential of graphene derivatives in periodontal tissue regeneration and engineering.

Material	Effect	References
rGO/HA nanocomposites	Increased ALP; mineralization; and osteopontin; osteocalcin expression	Lee et al., 2015 [182]
Poly(L-lactic-co-glycolic acid) with Tussah silk fibroin; GO	High adhesion; proliferation; ALP; mineral deposition	Shao et al., 2016 [183]
Silk-fibroin/GO	Osteogenic and cementoblast differentiation	Vera-Sánchez et al., 2016 [173]
CaP/rGO	Accelerated bone neo-formation	Kim et al., 2017 [184]
Collagen-GO membrane	Roughness and stiffness; osteogenic differentiation	Marco et al. 2017 [158]
Ti/GO/BMP-2/vancomycin	Osteogenic activity	Han et al., 2018 [185]
3D collagen sponge/GO	Osteogenic differentiation; PDL-like and cementum-like tissue regeneration	Kawamoto et al., 2018 [179]
monocytes activator GO complexed with CaP	Activation of monocytes; stimulated osteogenesis	Bordoni et al., 2019 [186]
GO-collagen aerogel	Biomineralization; biocompatibility; osteogenic activity	Liu et al., 2019 [187]
HA/rGO	Proliferation; osteogenic activity	Zhou et al., 2019 [188]
Silk fibrinoid/GO/BMP-2	Biocompatibility; adhesion; proliferation; osteogenic differentiation	Wu et al., 2019 [189]
GO/chitosan	Osteogenic differentiation	Amiryaghoubi et al. (2020) [190]
GO/IONPs/H	Biocompatible; osteogenic activity; calcium deposits	Pathmanapan et al., 2020 [191]
GO/HA/Au	Biocompatibility; osteogenic differentiation	Prakash et al., 2020 [192]

ALP: alkaline phosphatase; Au: gold; BMP-2: bone morphogenetic protein-2; CaP: calcium phosphate; GO: graphene oxide; HA: hydroxyapatite; IONPs: iron oxide nanoparticles; nHA: nanohydroxyapatite; PDL: periodontal ligament; rGO: reduced graphene oxide.

**Table 5:** Applications of graphene-based materials in direct and indirect restorations.

Material	Effect	References
PMMA/rGO—incorporated into the liquid	High concentrations decreased PMMA tensile strength. Lower concentrations exhibited no changes	Tripathi et al., 2013 [226]
Gp-NSs	Improved physical-mechanical properties of bioactive cement	Dubey et al., 2017 [193]
nHA/MWCNTO/GO	Formation of a protective layer for dentin against erosive processes	Nahorny et al., 2017 [201]
rGO-HA	The elasticity has improved tenfold compared with that of HA	Rajesh et al., 2017 [227]
GO-based fluorhydroxyapatite	Enamel and dentin mineralization	Shi et al., 2017 [228]
PMMA/GO—incorporated into the liquid	GO-concentrations ≥ 0.5 wt% increased the PMMA hardness and flexural strength	Lee et al., 2018 [216]
Fluorinated graphene	Increased microhardness and compressive strength; decreased friction coefficient	Sun et al., 2018 [195]
PMMA/GO—Commercial CAD-CAM resin block	GO incorporation into PMMA did not influence the hardness or flexural strength	Agarwalla et al., 2019 [40]
G/AgNp	Minimal toxicity and improved flexural properties	Bacali et al., 2019 [219]
GO	Enhanced shear bond strength	Khan et al., 2019 [152]
Graphite Fluoride bioactive glass	Enamel and dentin mineralization	Nam et al., 2019 [229]
PMMA/GO—Commercial CAD-CAM resin block	Increased flexural strength	Di Carlo et al., 2020 [230]
PMMA/GO—incorporated into the liquid	Decreased flexural strength	Ghosh and Shetty, 2020 [231]
PEEK/GNP—injection molding	Higher flexural, tensile, and compression strength	Jiang et al., 2021 [232]
Bone cement PMMA based/GO incorporated into the liquid	Increased bone cement compression strength	Levenez et al., 2021 [233]
PMMA/GO—Commercial CAD-CAM resin block	Decreased hardness	Ciocan et al., 2021 [234]
PMMA/GO—Commercial CAD-CAM resin block	Increased flexural strength	Çakmak et al., 2022 [235]
PMMA/GNP—3D printed resin	Improved strength, hardness, and elasticity; antimicrobial activity	Aati et al., 2022 [236]
Soft denture liner PMMA based/GO—incorporated into the liquid	No influence on denture liner hardness	Khan et al., 2022 [237]
GO/montmorillonite	A more stable compound; enamel and dentin mineralization	Velo et al., 2022 [238]

CAD/CAM: computer-aided design/computer-aided manufacturing; G/AgNP: graphene silver nanoparticles; GNP: Graphene nanoplatelets; GO: graphene oxide; Gp-NSs: Multilayer graphene nanosheets; HA: hydroxyapatite; nHA/MWCNTO/GO: Multiwalled carbon nanotube/graphene oxide hybrid carbon-based material combined with nanohydroxyapatite; PEEK: Poly-ether-ether-ketone; PMMA: Polymethylmethacrylate; rGO: reduced graphene oxide.

## Data Availability

Not applicable.
